# Immunohistochemical expression of promyelocytic leukemia body in soft tissue sarcomas

**DOI:** 10.1186/1756-9966-27-73

**Published:** 2008-11-23

**Authors:** Toshihiro Matsuo, Takashi Sugita, Shoji Shimose, Tadahiko Kubo, Masataka Ishikawa, Yuji Yasunaga, Mitsuo Ochi

**Affiliations:** 1 Department of Artificial Joints and Biomaterials, Graduate School of Biomedical Sciences, Hiroshima University, 1-2-3 Kasumi, Minami-ku, Hiroshima 734-8551, Japan; 2 Department of Orthopaedic Surgery, Hiroshima Prefectural Hospital, 1-5-54 Ujinakanda, Minami-ku, Hiroshima 734-8530, Japan; 3 Department of Orthopaedic Surgery, Graduate School of Biomedical Sciences, Hiroshima University, 1-2-3 Kasumi, Minami-ku, Hiroshima 734-8551, Japan

## Abstract

**Background:**

The function of promyelocytic leukemia (PML) bodies is not well known but plays an important role in controlling cell proliferation, apoptosis and senescence. This study was undertaken to analyze the clinical significance of PML body expression in primary tumor samples from malignant fibrous histiocytoma (MFH) and liposarcoma patients.

**Methods:**

We studied MFH and liposarcoma samples from 55 patients for PML bodies. Fluorescent immunostaining of PML bodies was performed in the paraffin-embedded tumor sections.

**Results:**

PML body immunostaining was identified in 63.9% of MFH and 63.2% of liposarcoma samples. PML body expression rates of all sarcoma cells were 1.5 ± 1.8% (range: 0–7.0) in MFH and 1.3 ± 1.4% (0–5.2) in liposarcoma samples. PML body expression (p = 0.0053) and a high rate of PML body expression (p = 0.0012) were significantly greater prognostic risk factors for death than the other clinical factors in MFH patients. All liposarcoma patients without expression of PML were disease free at the end of the study.

**Conclusion:**

Our study suggests that the presence of PML bodies may indicate a poor prognosis for MFH and liposarcoma patients.

## Background

PML was originally identified in leukemic blasts from acute promyelocytic leukemia (APL) patients, and may play a role in leukemogenesis [1-3]. PML bodies are donut-shaped nuclear domains containing PML protein [4], and are dynamic structures present in many normal and neoplastic tissues [5]. PML bodies also seem to play a multifaceted role in various cellular processes, including cell proliferation [6,7], cellular senescence [8-10], apoptosis [11-13], and tumor suppression [14,15] so that the function of PML bodies may well be an important contributing factor in the pathogenesis of malignant tumors. However, little is known concerning expression of PML bodies in sarcomas, including whether or not expression can be used as a prognostic indicator of sarcomas. We present the results of an analysis of the clinical significance of PML body expression in primary tumor samples from malignant fibrous histiocytoma and liposarcoma patients.

## Patients and methods

Among the patients who underwent surgery between 1989 and 2003, a total of 55 (36 MFHs and 19 liposarcomas) soft tissue sarcoma samples were obtained at the time of surgery. Informed consent was obtained from patients before surgery. The samples were fixed in 10% formalin and embedded into paraffin for immunohistochemistry. All clinical data are shown in Tables 1 and 2. We collected all primary tumor samples by biopsy or resection, and no patients had undergone chemotherapy before surgical specimens were collected. Induction chemotherapy was not used in any MFH and liposarcoma patients. The tumor size was evaluated by measurement of the largest diameter on MR images. Histological grades were assigned according to the French Federation of Cancer Centers Sarcoma Group (FNCLCC) system based on tumor differentiation, mitotic count and necrosis [16]. Surgical margins achieved were classified using the method described previously [17]. We performed brachytherapy or external radiation therapy following conservative surgery for all patients who received marginal resection.

**Table 1:** Data of 36 patients with soft tissue MFH

**No**.	**Age (yrs)**	**Gender**	**Site**	**Histological subtype**	**Histological grade**	**Tumor size (cm)**	**Surgical margin**	**Recurrence**	**Metastasis**	**Prognosis**	**Period (months)**	**PML (%)**	**PML expression**
1	53	male	thigh	stori-pleo	3	10	wide	-	+	DOD	12	1.2	+

2	48	male	thigh	myxoid	3	13	marginal	+	-	NED	80	0.0	-

3	76	female	thigh	stori-pleo	3	6.5	wide	-	+	DOD	22	2.2	+

4	54	male	thigh	stori-pleo	3	9	wide	-	+	DOD	12	0.5	+

5	49	male	upperarm	stori-pleo	3	10	marginal	-	+	DOD	18	4.2	+

6	63	female	axillar	myxoid	2	4	wide	-	-	CDF	28	2.0	+

7	82	male	thigh	stori-pleo	3	10.5	marginal	-	-	CDF	80	1.0	+

8	66	female	thigh	stori-pleo	2	6	marginal	-	-	CDF	60	0.0	-

9	75	male	thigh	stori-pleo	2	13	wide	+	-	NED	35	1.7	+

10	45	female	inguinal	myxoid	1	7	marginal	-	-	CDF	27	0.0	-

11	71	female	lower leg	myxoid	2	12	wide	+	+	DOD	58	3.4	+

12	74	female	thigh	myxoid	2	4.5	wide	-	-	CDF	73	0.4	+

13	53	male	lower leg	stori-pleo	3	10	wide	-	-	CDF	30	0.0	-

14	78	female	thigh	stori-pleo	2	9	marginal	-	+	DOD	9	2.0	+

15	35	male	thigh	stori-pleo	2	9	wide	-	-	CDF	52	0.0	-

16	81	male	thigh	stori-pleo	3	8	wide	-	-	CDF	26	2.0	+

17	84	male	buttock	stori-pleo	2	7.5	marginal	-	-	CDF	26	0.0	-

18	57	female	shoulder	stori-pleo	2	5	wide	-	-	CDF	62	0.0	-

19	76	female	thigh	stori-pleo	2	14	wide	-	+	DOD	6	1.8	+

20	75	male	thigh	stori-pleo	3	8	wide	-	+	DOD	10	3.0	+

21	57	male	thigh	stori-pleo	2	8.5	wide	-	-	CDF	94	1.6	+

22	72	male	thigh	stori-pleo	2	15	marginal	-	+	DOD	49	0.5	+

23	64	female	buttock	myxoid	3	11	marginal	-	+	DOD	10	2.8	+

24	55	female	thigh	myxoid	2	8	wide	-	+	DOD	21	7.0	+

25	59	female	shoulder	stori-pleo	2	12	marginal	+	+	DOD	47	6.4	+

26	74	male	thigh	myxoid	2	9	wide	-	+	DOD	27	0.0	-

27	46	male	thigh	stori-pleo	3	5.5	wide	-	-	CDF	98	2.0	+

28	73	male	thigh	stori-pleo	2	5.5	wide	-	-	CDF	112	1.0	+

29	62	female	forearm	myxoid	3	10	wide	-	-	CDF	138	0.0	-

30	49	male	upperarm	stori-pleo	2	5.5	wide	-	-	CDF	87	1.0	+

31	85	male	thigh	stori-pleo	3	11.5	marginal	-	-	CDF	106	0.0	-

32	58	female	buttock	stori-pleo	3	10.5	marginal	+	+	DOD	6	3.4	+

33	73	male	thigh	stori-pleo	2	6	wide	-	-	CDF	112	0.0	-

34	71	female	lower leg	myxoid	2	12	marginal	+	-	NED	65	0.0	-

35	73	female	lower leg	myxoid	2	7	wide	-	-	CDF	25	1.2	+

36	45	female	thigh	myxoid	2	6.5	marginal	-	-	CDF	29	0.0	-

stori-pleo: storiform-pleomorphic

CDF: continuous disease free

NED: no evidence of disease

DOD: dead of disease

**Table 2:** Data of 19 patients with liposarcoma

**No**.	**Age (yrs)**	**Gender**	**Site**	**Histological subtype**	**Histological grade**	**Tumor size (cm)**	**Surgical margin**	**Recurrence**	**Metastasis**	**Prognosis**	**Period (months)**	**PML (%)**	**PML expression**
1	35	female	popliteal	myxoid	2	14	marginal	-	-	CDF	65	0.0	-

2	50	female	thigh	myxoid	3	6	wide	-	-	CDF	55	2.2	+

3	48	female	forearm	myxoid	2	8	marginal	-	-	CDF	12	3.0	+

4	66	female	lower leg	dedifferentiated	3	8	marginal	-	-	CDF	56	0.0	-

5	66	male	upper arm	myxoid	3	11	wide	-	-	CDF	40	0.0	-

6	60	male	thigh	myxoid	2	16	wide	-	-	CDF	47	1.4	+

7	74	male	thigh	myxoid	3	8	wide	-	+	DOD	27	1.2	+

8	60	male	thigh	pleomorphic	3	10	wide	-	-	CDF	132	0.0	-

9	51	male	thigh	round cell	3	9	wide	-	+	DOD	12	2.1	+

10	66	male	shoulder	myxoid	2	8	wide	-	-	CDF	70	2.0	+

11	68	male	thigh	myxoid	3	12	marginal	-	-	CDF	120	0.0	-

12	43	female	thigh	myxoid	2	8	wide	-	-	CDF	60	0.0	-

13	47	male	forearm	dedifferentiated	3	11	marginal	-	+	DOD	12	1.2	+

14	62	female	thigh	myxoid	2	15	wide	-	-	CDF	62	1.7	+

15	67	female	thigh	myxoid	2	15	wide	-	-	CDF	60	0.4	+

16	63	male	thigh	pleomorphic	2	12	marginal	-	-	CDF	54	2.0	+

17	73	female	buttock	myxoid	2	8	wide	-	-	CDF	65	0.0	-

18	52	female	thigh	myxoid	3	6	wide	-	-	CDF	49	1.5	+

19	48	male	thigh	myxoid	2	20	marginal	+	+	DOD	15	5.2	+

### PML body immunofluorescence

Immunostaining was performed on the paraffin-embedded tumor sections. In short, the paraffin block was cut into 8 um sections and placed onto slides, followed by deparaffinization in xylene, then rehydration in alcohol. The next step was to place them in 10 mmol of 80 degree preheated Na citrate for 30 min. The slides were cooled, rinsed in PBS, and ten percent BSA was used to block cells. The slides were then incubated in the primary PML antibody, mouse monoclonal PML (PG-M3; Santa Cruz Biotechnology, Inc., Santa Cruz, CA) (1:100 dilution) overnight. Primary antibody was detected with FITC-conjugated gout anti-mouse IgG (1:200 dilution). Finally, the slides were washed in PBS and mounted in vectashield with DAPI.

### Immunohistochemical evaluation

Two independent, blind observers evaluated immunostained sections and the rates of stained cells were determined based on average values. For the evaluation of immunostained cells, we examined at least 700 sarcoma cells to determine whether their nuclei were positive for PML body staining. For all analysis, the samples in which stained cells made up 0.3% of the cells were regarded as positive.

### Statistical analysis

The cumulative prospective of overall survival was calculated using the method of Kaplan-Meier. Statistical significance of the differences between the survival curves was evaluated using the log-rank test. Each prognostic factor was divided into two groups based on an average value. Data are presented as the mean ± SD. In all analyses, a p value of < 0.05 was considered to indicate significance. All analyses were performed by statistical package Statview, Version 5.0 (Abacus Concepts, Berkley, CA).

## Results

**Figure 1:**
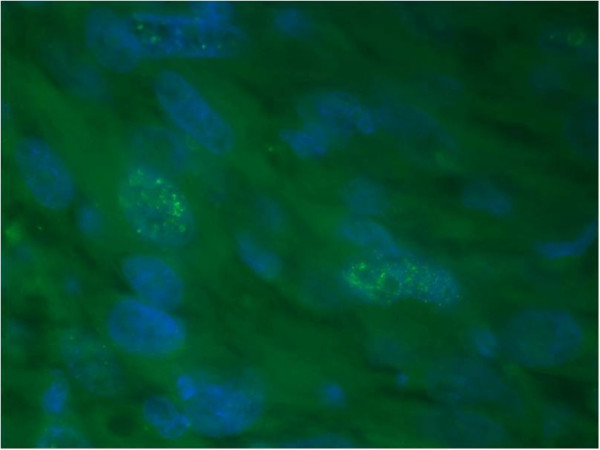
PML immunostaining on paraffin sections of MFH (patient No.23) × 1000.

**Figure 2:**
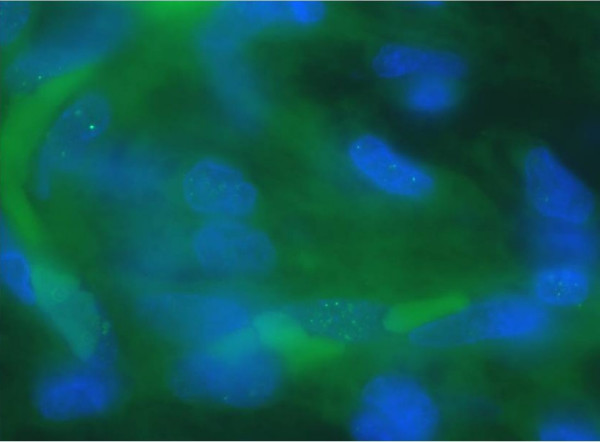
PML immunostaining on paraffin sections of liposarcoma (patient No.7) × 1000.

Immunofluorescence of PML body immunostaining on paraffin sections identified 23 of 36 tumors (63.9%) in MFHs and 12 of 19 (63.2%) in liposarcomas. PML body expression rates of all sarcoma cells were 1.5 ± 1.8% (range: 0–7.0) in MFHs and 1.3 ± 1.4% (0–5.2) in liposarcomas (Figures 1 and 2, Tables 1 and 2).

### Prognostic factors

#### MFH patients

**Figure 3:**
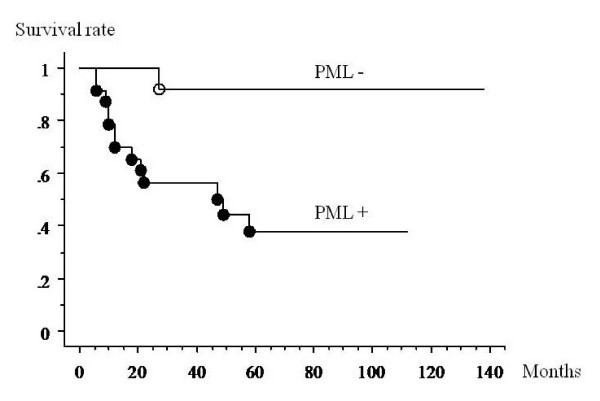
**Kaplan-Meier analysis of the association between survival and the presence of PML expression in MFH samples**. The patients with PML body expression had a worse prognosis than those who did not (p = 0.0053).

**Figure 4:**
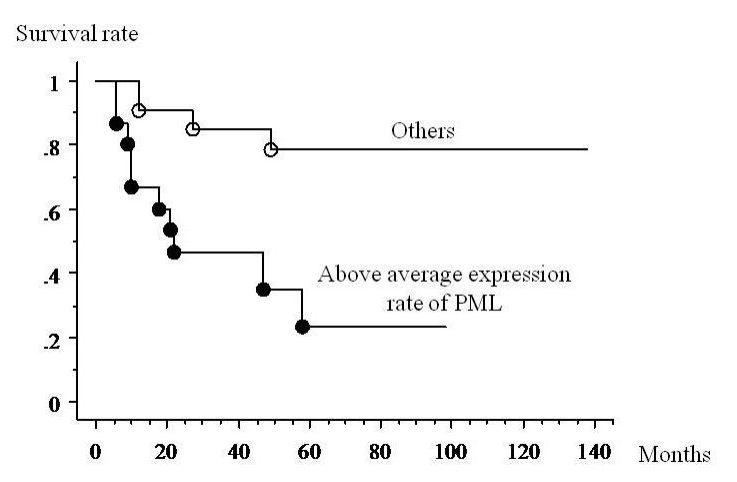
**Kaplan-Meier analysis of the association between survival and the rate of PML expression in MFH samples**. Patients who had a higher than average expression rate of PML bodies had a worse prognosis than other patients (p = 0.0012).

With univariate analysis, PML body expression and a high rate of PML body expression were significant prognostic risk factors for death. The patients with PML body expression had a worse prognosis than those who did not (p = 0.0053) (Figure 3). Patients who had a higher than average expression rate of PML bodies had a worse prognosis than other patients (p = 0.0012) (Figure 4). There were no significant differences between the survival rate and other factors (age: p = 0.919; gender: p = 0.297; histological grade: p = 0.204; tumor size: p = 0.198; surgical margin: p = 0.672; recurrence: p = 0.723).

#### Liposarcoma patients

**Figure 5:**
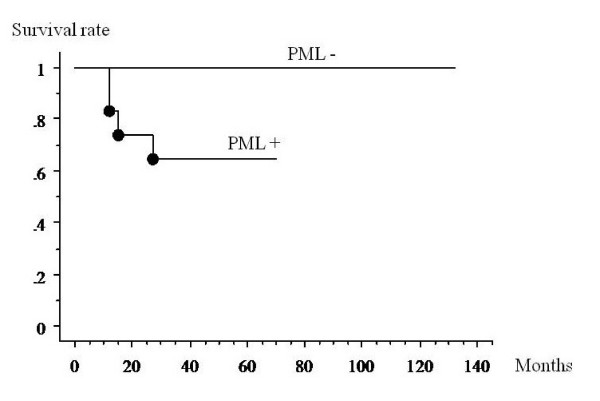
**Kaplan-Meier analysis of the association between survival and the expression of PML in liposarcoma samples.** All liposarcoma patients who had no expression of PML bodies were disease free.

All liposarcoma patients who had no PML body expression were disease free (Figure 5). The patients with local recurrence had a worse prognosis than those who did not (p = 0.0332). There were no significant differences between patients who had a higher than verage expression rate of PML bodies than other patients (p = 0.826). There were no significant differences between the survival rate and other factors (age: p = 0.0907; histological grade: p = 0.243; tumor size: p = 0.880; surgical margin: p = 0.458; high rate of PML body expression: p = 0.826).

## Discussion

PML protein is the product of the PML gene. It forms a fusion protein with the retinoic acid receptor (RAR)-α due to chromosome 15; 17 translocation in acute promyelocytic leukemia (APL) [1-3,18]. It is highly likely that PML protein plays a role in the regulation of transcriptional activity [19]. Several papers have revealed the probability that the PML/RAR-α fusion protein plays a key role in the pathogenesis of APL by altering the structure of the PML bodies [19-23]. The PML bodies are present in many kinds of tissues but not in all normal and tumor tissues [5]. It has become apparent that PML is an important contributing factor in the pathogenesis of malignant tumors [24]. Terris et al. reported that PML bodies are not restricted to APL but may be extended to other types of tumor and may be linked to cell proliferation [25]. In our present study, PML body immunostaining was identified in 23 of 36 MFHs (63.9%) and in 12 of 19 (63.2%) liposarcomas. Therefore, sarcomas also may influence PML bodies and PML may play an important role for tumorigenesis in sarcomas.

In our study, PML body expression (p = 0.0053) and a high rate of PML body expression (p = 0.0012) were significant prognostic risk factors for death in MFH patients, and all liposarcoma patients without expression of PML were alive at the end of the study. The prognosis for patients with sarcomas depends on the histological grade, tumor size and local recurrence [26]. However, in our present study, the PML body expression for patients correlates more strongly with a worse prognosis than these clinical factors, so that the presence of PML bodies indicated the likelihood of poor outcomes in patients with soft tissue sarcomas.

Several studies indicate that overexpression of PML protein inhibits cell cycle progression leading to G1/S arrest, suppresses some forms of oncogenic transformation and promotes apoptosis [27-29]. Also, recent study suggests that the PML protein may play a critical role in the suppression of angiogenesis in tumors [30]. However, in contrast, some report suggests that increased expression of PML was observed in several carcinoma cells and might be functionally related to cellular growth [25], indicating all PML will not be functionally equivalent in various cases [27]. The up-regulation of PML could be secondary to the release of several cytokines [25]. In fact MFH cells secrete high levels of IL-6 and elevation of the IL-6 occurs on tumor recurrence [31,32]. Strong expression of the tumor associated IL-6 was confirmed in liposarcoma cells [33]. Therefore, we hypothesize that increased expression of PML in sarcomas may be secondary to the secretion of cytokines and relate to cellular growth and proliferation.

There are two telomere maintenance mechanisms in human tumors: telomerase activity and alternative lengthening of telomeres (ALT) [34,35]. Some immortalized telomerase-negative cell lines possess extremely long and heterogeneous telomeres as an ALT, and a substantial proportion of types of sarcomas have been reported to have ALT without telomerase activity [36,37]. ALT cells are characterized by ALT-associated PML bodies (APBs), and APB is reported as a simple hallmark of ALT, although some report suggests that APBs are not always essential for ALT [38]. Several papers have revealed that ALT is a significant prognostic factor in sarcoma patients [39-41]. Although the expression of PML is not directly associated with APB, the association of the ALT with the PML may influence the prognosis of sarcomas. Our results are based on a small number of samples, so additional research of PML functions for sarcomas is warranted.

In summary, PML bodies may be a useful objective marker in the assessment of sarcoma prognosis. Due to the fact that more than 60% of sarcomas have PML body expression, PML may be associated with pathogenesis and/or tumor behavior of sarcomas.

## Competing interests

The authors declare that they have no competing interests.

## Authors' contributions

TM designed this study, collected the data, performed the experimental work and drafted the manuscript. TS collected the data, participated in the design of this study, and modified the manuscript. SS collected the data and modified the manuscript. TK and MI analysed the immunohistochemical results. YY and MO participated in the design of this study and modified the manuscript.

## References

[B1] Zhu J, Lallemand-Breitenbach V, de Thé H (2001). Pathways of retinoic acid- or arsenic trioxide-induced PML/RARalpha catabolism, role of oncogene degradation in disease remission. Oncogene.

[B2] de Thé H, Chomienne C, Lanotte M, Degos L, Dejean A (1990). The t(15;17) translocation of acute promyelocytic leukaemia fuses the retinoic acid receptor alpha gene to a novel transcribed locus. Nature.

[B3] Kakizuka A, Miller WH, Umesono K, Warrell RP, Frankel SR, Murty VV, Dmitrovsky E, Evans RM (1991). Chromosomal translocation t(15;17) in human acute promyelocytic leukemia fuses RAR alpha with a novel putative transcription factor, PML. Cell.

[B4] Hodges M, Tissot C, Howe K, Grimwade D, Freemont PS (1998). Structure, organization, and dynamics of promyelocytic leukemia protein nuclear bodies. Am J Hum Genet.

[B5] Gambacorta M, Flenghi L, Fagioli M, Pileri S, Leoncini L, Bigerna B, Pacini R, Tanci LN, Pasqualucci L, Ascani S, Mencarelli A, Liso A, Pelicci PG, Falini B (1996). Heterogeneous nuclear expression of the promyelocytic leukemia (PML) protein in normal and neoplastic human tissues. Am J Pathol.

[B6] Fagioli M, Alcalay M, Tomassoni L, Ferrucci PF, Mencarelli A, Riganelli D, Grignani F, Pozzan T, Nicoletti I, Grignani F, Pelicci PG (1998). Cooperation between the RING + B1-B2 and coiled-coil domains of PML is necessary for its effects on cell survival. Oncogene.

[B7] Cohen N, Sharma M, Kentsis A, Perez JM, Strudwick S, Borden KL (2001). PML RING suppresses oncogenic transformation by reducing the affinity of eIF4E for mRNA. EMBO J.

[B8] Pearson M, Carbone R, Sebastiani C, Cioce M, Fagioli M, Saito S, Higashimoto Y, Appella E, Minucci S, Pandolfi PP, Pelicci PG (2000). PML regulates p53 acetylation and premature senescence induced by oncogenic Ras. Nature.

[B9] Ferbeyre G, de Stanchina E, Querido E, Baptiste N, Prives C, Lowe SW (2000). PML is induced by oncogenic ras and promotes premature senescence. Genes Dev.

[B10] Bischof O, Kirsh O, Pearson M, Itahana K, Pelicci PG, Dejean A (2002). Deconstructing PML-induced premature senescence. EMBO J.

[B11] Wang ZG, Ruggero D, Ronchetti S, Zhong S, Gaboli M, Rivi R, Pandolfi PP (1998). PML is essential for multiple apoptotic pathways. Nat Genet.

[B12] Fogal V, Gostissa M, Sandy P, Zacchi P, Sternsdorf T, Jensen K, Pandolfi PP, Will H, Schneider C, Del Sal G (2000). Regulation of p53 activity in nuclear bodies by a specific PML isoform. EMBO J.

[B13] Guo A, Salomoni P, Luo J, Shih A, Zhong S, Gu W, Pandolfi PP (2000). The function of PML in p53-dependent apoptosis. Nat Cell Biol.

[B14] Salomoni P, Pandolfi PP (2002). The role of PML in tumor suppression. Cell.

[B15] Hofmann TG, Will H (2003). Body language: the function of PML nuclear bodies in apoptosis regulation. Cell Death Differ.

[B16] Guillou L, Coindre JM, Bonichon F, Nguyen BB, Terrier P, Collin F, Vilain MO, Mandard AM, Le Doussal V, Leroux A, Jacquemier J, Duplay H, Sastre-Garau X, Costa J (1997). Comparative study of the National Cancer Institute and French Federation of Cancer Centers Sarcoma Group grading systems in a population of 410 adult patients with soft tissue sarcoma. J Clin Oncol.

[B17] Kawaguchi N, Matumoto S, Manabe J (1995). New method of evaluating the surgical margin and safety margin for musculoskeletal sarcoma, analysed on the basis of 457 surgical cases. J Cancer Res Clin Oncol.

[B18] Melnick A, Licht JD (1999). Deconstructing a disease: RARalpha, its fusion partners, and their roles in the pathogenesis of acute promyelocytic leukemia. Blood.

[B19] Grignani F, Fagioli M, Alcalay M, Longo L, Pandolfi PP, Donti E, Biondi A, Lo Coco F, Grignani F, Pelicci PG (1994). Acute promyelocytic leukemia: from genetics to treatment. Blood.

[B20] Warrell RP, de Thé H, Wang ZY, Degos L (1993). Acute promyelocytic leukemia. N Engl J Med.

[B21] de Thé H, Lavau C, Marchio A, Chomienne C, Degos L, Dejean A (1991). The PML-RAR alpha fusion mRNA generated by the t(15;17) translocation in acute promyelocytic leukemia encodes a functionally altered RAR. Cell.

[B22] Pandolfi PP, Alcalay M, Fagioli M, Zangrilli D, Mencarelli A, Diverio D, Biondi A, Lo Coco F, Rambaldi A, Grignani F (1992). Genomic variability and alternative splicing generate multiple PML/RAR alpha transcripts that encode aberrant PML proteins and PML/RAR alpha isoforms in acute promyelocytic leukaemia. EMBO J.

[B23] Alcalay M, Zangrilli D, Fagioli M, Pandolfi PP, Mencarelli A, Lo Coco F, Biondi A, Grignani F, Pelicci PG (1992). Expression pattern of the RAR alpha-PML fusion gene in acute promyelocytic leukemia. Proc Natl Acad Sci USA.

[B24] Salomoni P, Ferguson BJ, Wyllie AH, Rich T (2008). New insights into the role of PML in tumour suppression. Cell Res.

[B25] Terris B, Baldin V, Dubois S, Degott C, Flejou JF, Hénin D, Dejean A (1995). PML nuclear bodies are general targets for inflammation and cell proliferation. Cancer Res.

[B26] Pisters PW, Leung DH, Woodruff J, Shi W, Brennan MF (1996). Analysis of prognostic factors in 1,041 patients with localized soft tissue sarcomas of the extremities. J Clin Oncol.

[B27] Borden KL (2002). Pondering the promyelocytic leukemia protein (PML) puzzle: possible functions for PML nuclear bodies. Mol Cell Biol.

[B28] Strudwick S, Borden KL (2002). The emerging roles of translation factor eIF4E in the nucleus. Differentiation.

[B29] Takahashi Y, Lallemand-Breitenbach V, Zhu J, de Thé H (2004). PML nuclear bodies and apoptosis. Oncogene.

[B30] Bernardi R, Guernah I, Jin D, Grisendi S, Alimonti A, Teruya-Feldstein J, Cordon-Cardo C, Simon MC, Rafii S, Pandolfi PP (2006). PML inhibits HIF-1alpha translation and neoangiogenesis through repression of mTOR. Nature.

[B31] Nakanishi H, Yoshioka K, Joyama S, Araki N, Myoui A, Ishiguro S, Ueda T, Yoshikawa H, Itoh K (2004). Interleukin-6/soluble interleukin-6 receptor signaling attenuates proliferation and invasion, and induces morphological changes of a newly established pleomorphic malignant fibrous histiocytoma cell line. Am J Pathol.

[B32] Shouda T, Hiraoka K, Komiya S, Hamada T, Zenmyo M, Iwasaki H, Isayama T, Fukushima N, Nagata K, Yoshimura A (2006). Suppression of IL-6 production and proliferation by blocking STAT3 activation in malignant soft tissue tumor cells. Cancer Lett.

[B33] Göransson M, Elias E, Ståhlberg A, Olofsson A, Andersson C, Aman P (2005). Myxoid liposarcoma FUS-DDIT3 fusion oncogene induces C/EBP beta-mediated interleukin 6 expression. Int J Cancer.

[B34] Shay JW, Wright WE (2002). Telomerase: a target for cancer therapeutics. Cancer Cell.

[B35] Reddel RR (2003). Alternative lengthening of telomeres, telomerase, and cancer. Cancer let.

[B36] Bryan TM, Englezou A, Dalla-Pozza L, Dunham MA, Reddel RR (1997). Evidence for an alternative mechanism for maintaining telomere length in human tumors and tumor-derived cell lines. Nat Med.

[B37] Bryan TM, Englezou A, Gupta J, Bacchetti S, Reddel RR (1995). Telomere elongation in immortal human cells without detectable telomerase activity. EMBO J.

[B38] Fasching CL, Bower K, Reddel RR (2005). Telomerase-independent telomere length maintenance in the absence of alternative lengthening of telomeres-associated promyelocytic leukemia bodies. Cancer Res.

[B39] Ulaner GA, Huang HY, Otero J, Zhao Z, Ben-Porat L, Satagopan JM, Gorlick R, Meyers P, Healey JH, Huvos AG, Hoffman AR, Ladanyi M (2003). Absence of a telomere maintenance mechanism as a favorable prognostic factor in patients with osteosarcoma. Cancer Res.

[B40] Ulaner GA, Hoffman AR, Otero J, Huang HY, Zhao Z, Mazumdar M, Gorlick R, Meyers P, Healey JH, Ladanyi M (2004). Divergent patterns of telomere maintenance mechanisms among human sarcomas: sharply contrasting prevalence of the alternative lengthening of telomeres mechanism in Ewing's sarcomas and osteosarcomas. Genes Chromosomes Cancer.

[B41] Henson JD, Hannay JA, McCarthy SW, Royds JA, Yeager TR, Robinson RA, Wharton SB, Jellinek DA, Arbuckle SM, Yoo J, Robinson BG, Learoyd DL, Stalley PD, Bonar SF, Yu D, Pollock RE, Reddel RR (2005). A robust assay for alternative lengthening of telomeres in tumors shows the significance of alternative lengthening of telomeres in sarcomas and astrocytomas. Clin Cancer Res.

